# Sowing Date Affects the Timing and Duration of Key Chickpea (*Cicer arietinum* L.) Growth Phases

**DOI:** 10.3390/plants9101257

**Published:** 2020-09-24

**Authors:** Mark F. Richards, Aaron L. Preston, Tony Napier, Leigh Jenkins, Lancelot Maphosa

**Affiliations:** 1 NSW Department of Primary Industries, 322 Pine Gully Road, Wagga Wagga, NSW 2650, Australia; aaron.preston@dpi.nsw.gov.au (A.L.P.); lance.maphosa@dpi.nsw.gov.au (L.M.); 2 NSW Department of Primary Industries, 2198 Irrigation Way, Yanco, NSW 2705, Australia; tony.napier@dpi.nsw.gov.au; 3 NSW Department of Primary Industries, 7878 Mitchell Highway, Trangie, NSW 2823, Australia; leigh.jenkins@dpi.nsw.gov.au

**Keywords:** abiotic, stresses, chickpea, flowering, genotype-by-environment, interactions, growing degree days, mega-environments, phenology, podding

## Abstract

Chickpea is the main legume rotation crop within farming systems in northern New South Wales (NSW), Australia, and is grown mainly under rainfed conditions. Recent expansion of chickpea growing areas in southern and central western NSW expose them to abiotic stresses; however, knowledge about how these stresses affect overall crop development is limited. This study aimed to examine the influence of sowing time on the timing and duration of key chickpea phenological growth phases in southern and central western environments of NSW. Experiments were conducted over two years in southern NSW (Leeton, Wagga Wagga and Yanco (one year)) and central western NSW (Trangie) to identify phenology responses. Climatic, phenology and experimental site data was recorded, and the duration of growth phases and growing degree days calculated. Early sowing (mid-April) generally delayed flowering, extending the crop’s vegetative period, and the progressive delay in sowing resulted in shorter vegetative and podding growth phases. All genotypes showed photoperiod sensitivity, and the mean daily temperature at sowing influenced time to emergence and to some extent crop establishment. This study concludes that environmental factors such as temperature, moisture availability and day length are the main drivers of phenological development in chickpea.

## 1. Introduction

Chickpea (*Cicer arietinum* L.) is a cool season legume grown mainly in arid and semi-arid regions, where its production is constrained by a range of environmental factors such as temperature, soil moisture availability and day length. In terms of production, it is the second most important pulse crop after common bean (*Phaseolus vulgaris* L.) with approximately 17.8 million ha grown across 56 countries [1]. Two types of chickpea are produced, *kabulis* which have a thin, white or cream coloured seed coat and white flowers, and *desis* which have a thick, tan to dark brown coloured seed coat, and purple coloured flowers. Generally, *desi* genotypes tend to display higher levels of drought and heat tolerance than *kabuli* genotypes [2].

Within farming systems, chickpea broadly constitutes a valuable break crop important for weed control, and prevention of soil and stubble borne cereal diseases through disrupting inoculum build up. Also, chickpea fixes atmospheric nitrogen and therefore improves soil fertility through retaining the residual nitrogen in the soil for the following cereal crop [3]. Depending on agronomy, precipitation, soil management and inoculation, under Australian conditions the amount of shoot nitrogen fixed ranges from 0–124 kg/ha, with an average of 40 kg/ha [4]. This residual nitrogen reduces production costs for subsequent crops in diversified farming enterprises. Chickpea’s nutritional and health benefits are well documented; it is a source of protein especially for vegetarians [5] and is also an important source of energy and protein in animal feed [6].

Crop plants have a range of avoidance or tolerance strategies in order to survive abiotic constraints [7]. Early phenology is a common crop strategy of escaping late season stresses and facilitates adaptation to short season environments. The plant growth duration must suit or complement a production environment and/or farming enterprise. At the genetic level, the genes controlling chickpea phenology and growth durations are largely characterised and are well known [2,8]. In addition to genetic knowledge, management practices such as optimising sowing dates for specific varieties can minimise the exposure to environmental stress factors during critical growth periods [9]. As such, using a range of sowing dates across diverse sites and years is a practical way of testing for adaptability in new regions, by matching performance of varieties to the long-term average climatic conditions.

Low temperatures tend to delay emergence, decrease plant growth rate and increase the growing degree days (GDD) required to switch from the vegetative phase to the reproductive phase. Minimum GDD thresholds have to be met for chickpea phenological development but the exact requirements vary with variety, environmental conditions and agronomic management practices such as sowing date and depth [10,11,12]. For example, there is accelerated development and shorter growth phases under late sowing and dry conditions [13,14]. The duration and rate of development can subsequently influence related traits such as biomass accumulation, rate of grain filling, harvest index and ultimately grain yield.

Although predominantly grown and consumed in the Indian subcontinent [2,15], chickpea production is increasingly expanding to new agro-ecological zones of Australia. In Australia, production is concentrated in the warm temperate north-east region (Northern New South Wales (NSW) and Queensland) and is expanding to the cooler temperate southern and central western regions of NSW. These southern and central western regions experience less favourable climatic conditions during the growing season such as water deficit and extreme high and low temperatures, limiting crop development, yield potential and adoption. Chickpeas in these environments are generally constrained by low temperatures early in the growing season and heat and moisture stress later in the season. These abiotic stresses can dramatically affect plant development and productivity [16] although the impact of these stresses on chickpea physiology in these environments is poorly understood.

The objectives of this study were to examine the influence of sowing time on the timing and duration of key chickpea phenological growth phases and characterise genotype crop phenology through understanding genotype-by-environment-by-management (G × E × M) interactions in southern and central western New South Wales. The study further quantifies the effects of GDD, sowing date, temperature, and photoperiod on chickpea phenology.

## 2. Results

### 2.1. Temperature at Sowing

Mean daily temperature, important for chickpea germination, varied between the sites (Table 1), progressively decreasing as sowing date was delayed and had a wider range in 2019 than 2018. At later sowing dates (SD3 and SD4), temperatures were higher at the central western site of Trangie than the southern sites.

### 2.2. Phenological Development

Supplementary Tables S1–S4 detail the results for individual experiments and show the effect of genotype, sowing date and their interactions across individual locations and years. Sowing date had no effect on establishment in 2018. However, in 2019 sowing date had different effects. For example, SD4 had the lowest establishment at WWAI but had the highest establishment at LFS. At TARC, in 2019, SD3 recorded the highest establishment. Time to emergence took longer at all sites when sowing was delayed, ranging from six days (SD1 at WWAI in 2018 and TARC in 2019) to 29 days (SD4 at LFS in 2019) (Table 2). Averaged across sowing dates, Genesis 090 was the first to emerge at both LFS and the nearby (6 km) YAI site, with PBA Striker emerging first at WWAI in 2018. Emergence was not recorded at TARC in 2018. In 2019, PBA Striker was first to emerge at LFS whereas Genesis 079 emerged earlier at WWAI. At TARC in 2019, emergence was similar for all genotypes except PBA Boundary and Genesis 090 which were slower to emerge. Generally, across sites and years, time from emergence to flowering, podding (10% and 50%) and physiological maturity was shorter when sowing was delayed (Table 2). As a result, the vegetative, flowering and podding phases as well as overall crop lifecycle were shorter in the later sown treatments. Interactions between genotype and sowing date were observed for some of the traits in all experiments (Supplementary Tables S1–S4).

### 2.3. Impact of Growing Degree Days (GDD) on Time to Flowering

**Figure 1:**
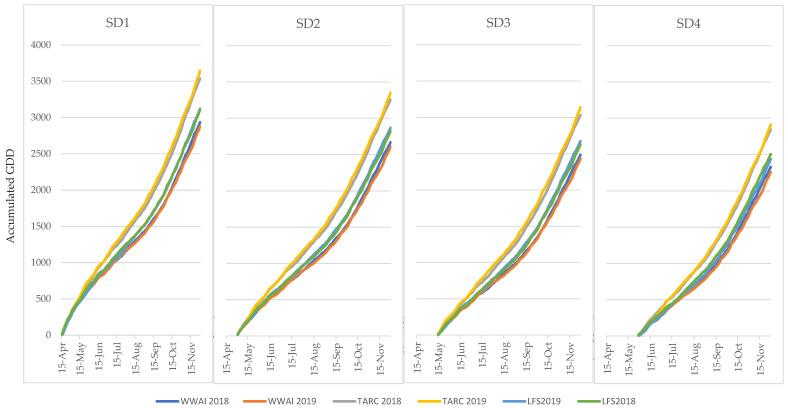
Rate of growing degree day accumulation over the growing season (April to November) at each location and year from each sowing date (SD1–4).

**Figure 2:**
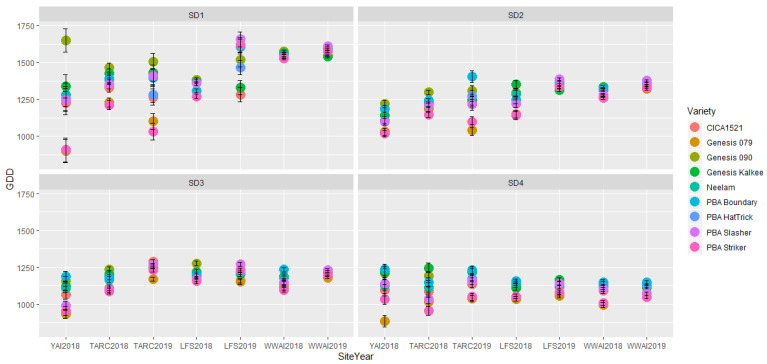
Accumulated growing degree days (GDD) for time to flowering at four sowing dates (SD) at Trangie (TARC), Wagga Wagga (WWAI), Leeton (LFS) and Yanco (YAI) during 2018 and 2019. In 2019, PBA HatTrick replaced Neelam. The l.s.d for G, SD and G × SD respectively are YAI2018: 52, 47, 104; TARC2018: 35, 18, 68; TARC2019: 54, 45, 109; LFS2018: 20, 19, 41; LFS2019: 26, 18, 52; WWAI2018: 9, 6, 17 and WWAI2019: 9, 6, 17.

The rate of accumulation of growing degree days was consistent at each site across both years (Figure 1). The northern site Trangie accumulated GDD quicker than Leeton and Wagga as expected, and Leeton was consistently warmer than Wagga across all SD treatments. The impact of GDD on time to flowering (Figure 2) followed the same trend at both WWAI and LFS in both years, with the crops requiring less GDD to flower as sowing date was delayed. At all the experiments, there were significant genotype, sowing date and G × SD interactions. The early maturing varieties, PBA Striker (desi) and Genesis 079 (kabuli) required lower GDD compared to the late maturing genotypes. Generally, the genotypes required more GDD at SD1 to reach flowering, with the GDD requirements progressively decreasing with delayed sowing. However, this overall trend was not observed at YAI in 2018 and TARC in 2019 (Figure 2). At YAI in 2018, both PBA Striker and Genesis 079 required less GDD at SD1 than at SD2, with SD2 requirements higher than all the other SDs. At TARC in 2019, both PBA Striker and Genesis 079 required more GDD to flower at SD3 than at the other sowing dates. Across all varieties, sites, sowing dates and years, the GDD accumulated for chickpea to flower ranged from 884 GDD for Genesis 079 at YAI in 2018, to 1658 GDD for PBA Slasher at LFS in 2019 (Figure 2). The range was wide at the drought stressed YAI18, with PBA Striker and Genesis 079 requiring low GDD, and Genesis 090 requiring very high GDD to be able to reach flowering. Variations were observed between years, especially in SD1, except in WWAI. At LFS in 2018, Genesis Kalkee was unique as it required similar GDD at SD1 and SD2, while at LFS in 2019, Genesis Kalkee, Genesis 079, and CICA1521 also had similar requirement at SD1 and SD2.

### 2.4. Environmental Correlations and Classification from the MET Analysis

**Figure 3:**
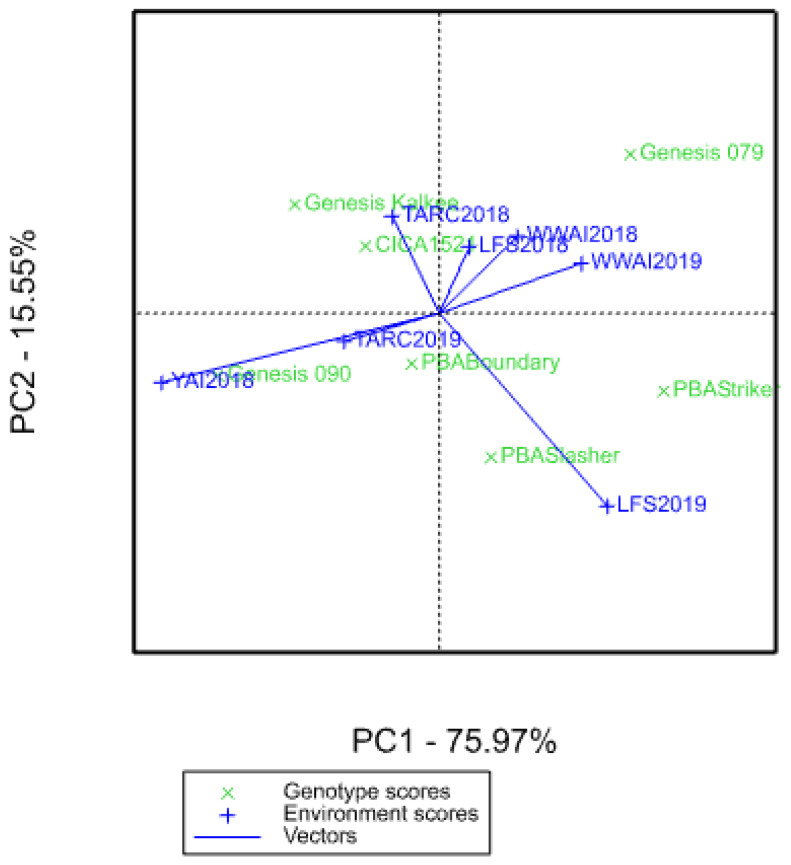
Additive main effects and multiplicative interaction (AMMI) biplot for days to flowering showing the correlation between environments and overall genotype stability and adaptability (at origin GEI = 0). Acute angle = positive correlation; right angle = no correlation, and obtuse angle = negative correlation.

The additive main effects and multiplicative interaction (AMMI) biplot visually represents genotype performance and environmental correlations for days to flowering. Both PCA1 (*p* < 0.001) and PCA2 (*p* = 0.019) are significant, with PC1 axis explaining 75.97% and PC2 axis 15.55% of the G × E interaction sum of squares (Figure 3). Therefore, the principal component axis explained 91.52% of the G × E interaction. The genotypes did not cluster together in the AMMI biplot. Genotypes PBA Boundary and CICA1521 are closer to the origin than PBA Slasher and Genesis 079. Environments LFS2018, WWAI2018 and WWAI2019 clustered together. There was a positive correlation between TARC2018 and LFS2018/WWAI2018, no correlation between TARC2018 and WWAI2019, YAI2018 and TARC2018, and negative correlation between TARC2018 and LFS2019. WWAI2018 and WWAI2019 were positively correlated, while there was no correlation between LFS2018 and LFS2019. The LFS experiments were negatively correlated with YAI2018. Environments YAI2018 and LFS2019 are further away from the origin.

**Figure 4:**
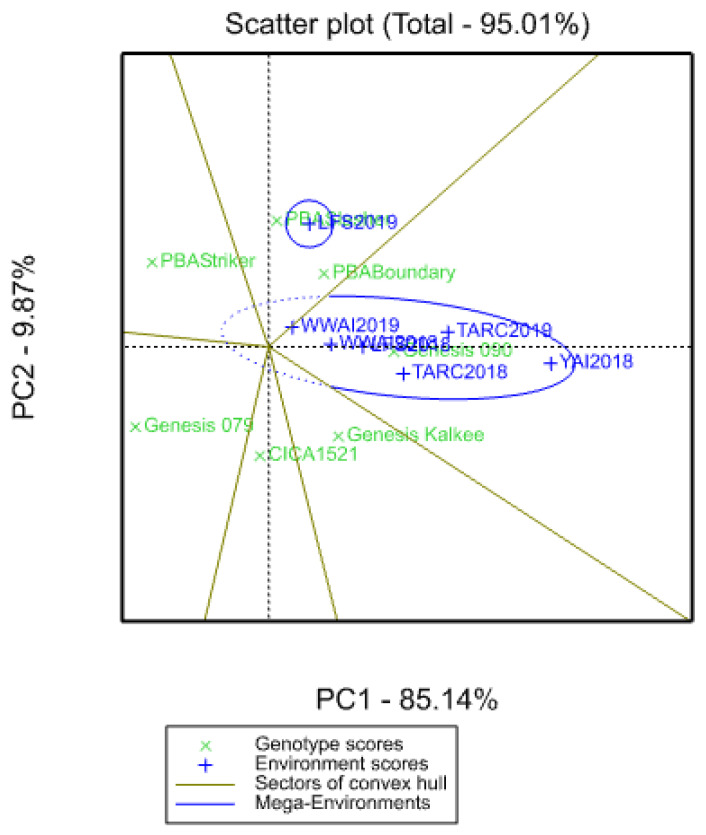
Genotype main effects (G) and genotype by environment interaction (G × E) (GGE) biplot for days to flowering showing different vectors and two mega-environments and genotype performance in the respective vectors and mega-environments.

The genotype main effects (G) and genotype by environment interaction (G × E) (GGE) biplot characterises the seven experiments into two mega-environments, one comprising LFS2019, and the other comprising the rest of the environments (Figure 4). Genotypes PBA Boundary and PBA Slasher took longer to flower in the sector comprising mega-environment LFS2019, while Genesis 090 was later to flower in the other environment. The remaining genotypes showed environmental sensitivity and were in different sectors.

## 3. Discussion

Despite the potential to be confounded by other environmental factors, examining the impact of sowing date on plant development remains a practical and inexpensive screening approach to test for adaptability of species to new production environments [13,17,18,19]. In the central western and southern environments of NSW, widening the chickpea sowing window from mid-April to the end of May, allowed testing of genotype, year, sowing date and location effects and proved to be an effective strategy to test chickpea phenology. Sowing date was shown to be a large source of variation to chickpea adaptation and overall phasic development in the individual experiments. Similar approaches have been used to identify the impact of abiotic stresses such as heat and drought amongst genotypes differing in maturity [14,17,18,19,20]. Using different sowing dates allows expression of varieties’ G × E interactions which supports agronomic guidelines for differing varieties to fit into preferred production and management systems. This ensures that phases such as flowering and podding that are critical to yield formation [9] are not adversely affected by environmental stress factors such as frost and late season heat and soil moisture stress. These environmental constraints drastically reduce chickpea productivity [21,22,23].

Growing degree days influenced the overall growth and phenological development. Emergence was delayed with later sowing, (e.g., SD3 and SD4) due to decreased soil temperature in late autumn (Table 1), requiring a longer time to satisfy the minimum GDD for emergence. Time to flowering was accelerated with later sowing and the accompanying slower accumulation of GDD. Delays in flowering have been shown to be negatively associated with grain yield in heat stressed experiments [17]. Generally, in early flowering genotypes the stored soil moisture is used during the reproductive phase as opposed to during the vegetative phase as is the case with late maturing genotypes. In the drought stressed YAI experiment, in 2018, the early maturing genotypes such as PBA Striker flowered at 1100 GDD, while at the nearby irrigated LFS experiment it flowered after accumulating over 1250 GDD (Figure 2). In addition to the 87 mm of in-season rainfall, the YAI experiment received 77 mm pre-sowing and 64 mm in-crop irrigation, compared to the LFS experiment which received 220 mm pre-sowing and 24 mm in-crop irrigation; the long-term average for growing season rainfall (April–October) for the region is 225 mm. At YAI, the pre-sowing irrigation was enough to enable emergence, but was insufficient to maintain crop growth throughout the season hence the need for supplementary in-crop irrigation.

Based on these GDD differences, we infer that in addition to GDD, other environmental factors such as soil moisture confound chickpea phenological development. Due to the dry conditions across all sowing times at YAI in 2018, we suspect that Genesis 090 is possibly a drought susceptible genotype as it showed signs of stress such as yellowing and crop wilting. Furthermore, the early sown (SD1) Genesis 090 initiated flowering as expected but due to the dry conditions its growth and/or development stopped temporarily, and it did not reach 50% flowering until the experiment was irrigated. This temporal stop in development may partly explain the very high “outlier” GDD required for this genotype to flower at SD1 (Figure 2). TARC is located at higher latitude and is therefore slightly warmer and has slightly longer daylength than in the southern sites during the growing season, which may partly explain the GDD requirement deviation of PBA Striker and Genesis 079 in 2019.

Plants sown at SD1 emerged when day length (photoperiod) was gradually decreasing (mid–late April). However, those sown on SD4 emerged when days were starting to lengthen after the winter solstice on 21 June. Chickpea can be photoperiod insensitive/day length neutral, intermediate sensitive or highly sensitive to day length [24]. The genotypes used in this study are all photoperiod sensitive as their ability to switch from the vegetative phase to the reproductive phase was affected by sowing time, with longer days reducing the thermal time target, thus requiring fewer GDD to flower. Similar crop behaviour was widely observed in cereals where photoperiod and vernalisation requirements control phenological development [25,26]. Like cereals, chickpea can require vernalisation before it is able to switch from the vegetative phase to the reproductive phase. These experiments were sown in autumn, going into winter, and it is most likely that the vernalisation requirements were met regardless of need. As such, photoperiod requirement would have been more limiting than the vernalisation requirement and this has been observed to be the case in winter sown wheat [27].

Late sowing and the accompanying increase in minimum and maximum temperatures accelerates plant growth rate and decreases days to physiological maturity [13,14] and was observed in this study.

As expected, the later sowings shortened the time to physiological maturity and the duration of vegetative, flowering and podding growth phases compared to earlier sowing dates (Table 2). At all sites and yearsthe duration of growth phases varied between sites, but were consistent for each site across years. Trangie had a shorter vegetative phase than the southern sites at Leeton and Wagga Wagga, with similar duration for all but SD3. Despite this, the flowering and podding phases were all significantly different with flowering and podding phase durations rapidly decreasing as sowing day was delayed. Leeton also had a shorter vegetative phase than Wagga Wagga, and a flowering duration similar to Trangie, but a podding phase duration similar to Wagga Wagga. This is consistent with the expectation of a GDD mediated shift from vegetative phase to reproductive phase occurring earlier at the warmer sites of Trangie and Leeton. This transition allowed a longer flowering period at these sites. Despite variation in vegetative and flowering phase duration, all plants across sowing dates and locations started podding when the mean daily temperature approached 15 °C.

In the AMMI model, environments LFS2018, WWAI2018, and WWAI2019 which are geographically closer, clustered together meaning they influence genotypes in a similar way (Figure 2.). However, they did not cluster with YAI2018 and LFS2019, which are also in southern NSW. In the drought stressed YAI2018, phenological development was quicker, a phenomenon commonly observed in chickpea [28,29]. Additionally, the early genotype PBA Striker matured significantly earlier at the YAI experiment than at the adjacent LFS experiment. Also, environment LFS2019 was an outlier when the experiments were clustered into mega-environments using the GGE model, while the other six fell into the same mega-environment. This is most likely a result of moderate levels of ascochyta blight observed early in the season but later contained by fungicides. The TARC environments had different watering regimes in the two years and distinctly stood out in the AMMI analysis but were in the same mega-environment.

Significant differences in crop phenology were observed between the genotypes at all the locations and years, and it has previously been shown that G × E interactions largely influence crop phenological development [30,31]. The seven genotypes common across all the experiments do not cluster together in the AMMI biplot (Figure 2) which means they behave differently across environments. PBA Boundary and CICA1521 are closer to the origin (which represents the mean of all the genotypes in all the environments) than PBA Slasher and Genesis 079. Genotypes closer to the origin are broadly adapted, do well across a wide range of conditions and are less sensitive to the environment. Genotypes further from the origin are adapted to more specific environments, for example PBA Slasher appears to be adapted to LFS2019. Specific genotypic adaptation could be beneficial as the genotype might be better able to exploit varying environmental conditions [32]. The mega-environments are a product of a combination of environmental factors that drive phenological development, beyond geographic location/soil characteristics which are peculiar to each site.

This study confirmed the major role of environmental conditions such as temperature, soil moisture availability, and day length as major drivers of chickpea phenological development and confirmed the diversity of the genotypes and their suitability to different environments. Abiotic stresses influenced chickpea phenological development leading to different GDD requirements for the genotypes used in this study. Low temperatures were observed to delay emergence (Table 1) and the rate of plant development. This ultimately lengthened the duration of the plant’s growing season, primarily by delaying flowering, leading to a longer vegetative phase (Table 2). The 2018 and 2019 seasons in southern and central western NSW were very challenging for growing pulse crops as they experienced low autumn and winter rainfall, and low temperatures early in the season. The below average seasonal rainfall across the two years identified genotype responses in dry seasons but not under ordinarily normal growing seasons. The impact of water stress on chickpea has been examined in other environments in Australia [33]; however, the specific effect in southern and central western NSW have not been quantified. Further research is required to account for moisture stress and the effect of weather conditions on each genotype relative to their phasic development. This is an important consideration to better understand genotype responses and validate their adaptation potential in each environment.

## 4. Materials and Methods

### 4.1. Experimental Locations and Management

**Figure 5:**
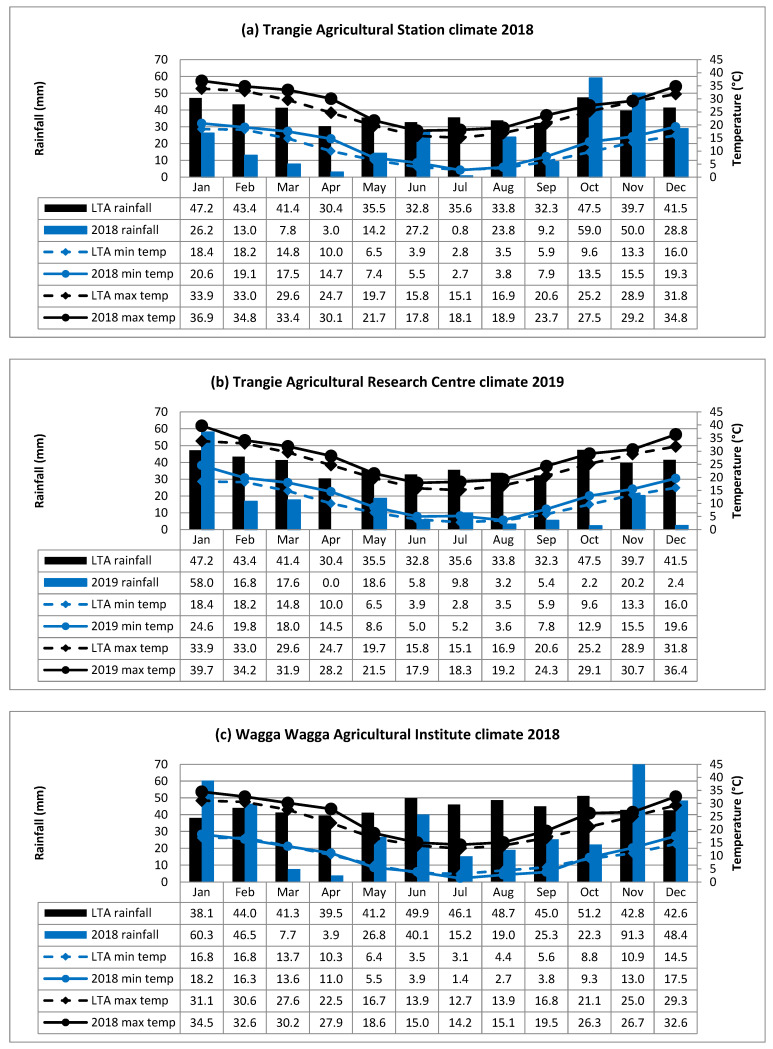

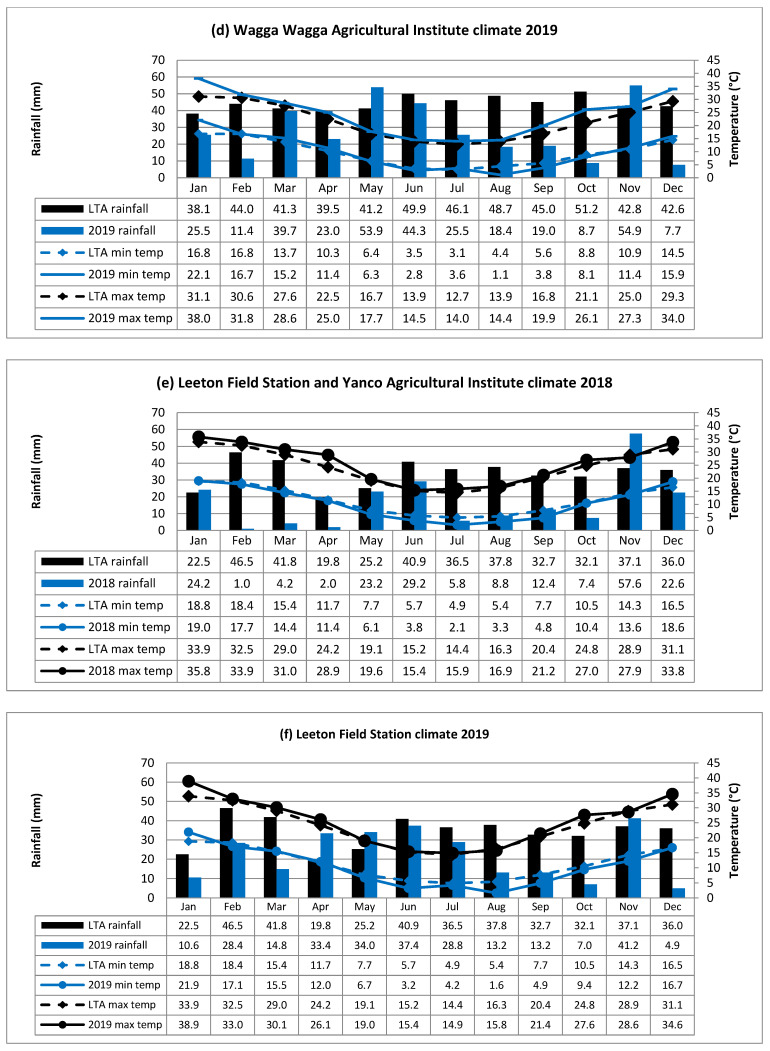
(**a**–**f**) Rainfall, minimum (min.) and maximum (max.) temperatures recorded in 2018 and 2019, together with the long-term averages (LTA) at TARC, WWAI, LFS, and YAI.

Experiments were conducted at Trangie in central western NSW and Wagga Wagga, Leeton and Yanco in southern NSW in 2018 and 2019 (Yanco 2018 only). Table 3 provides details of the experimental sites, seasonal rainfall, supplementary irrigation and overall management practices. Monthly rainfall, minimum and maximum temperatures for 2018 and 2019, together with long term site averages, obtained from the Australian Bureau of Meteorology (BOM) website (http://www.bom.gov.au), are shown in Figure 5a–f. In 2018, the experiments were conducted at all four locations: Trangie Agricultural Research Centre (TARC), Wagga Wagga Agricultural Institute (WWAI), Leeton Field Station (LFS) and Yanco Agricultural Institute (YAI). The YAI, which is nearby (6 km) to LFS was treated as a drought stressed experiment. The sowing times were similar at all sites, with fortnightly sowing from mid-April to end of May. These sowing times were selected to cover all likely sowing dates within, and slightly earlier than, the commercially recommended sowing window.

### 4.2. Plant Material and Experiment Conditions

In total, nine diverse chickpea genotypes (including both *desi* and *kabuli* types) consisting of released varieties and an advanced breeding line (CICA1521) (Table 4), were used in this study to evaluate phenological development, across four sowing dates. Seven genotypes were common across all sites in both years, as PBA HatTrick replaced Neelam in 2019. The genotypes were selected based on their diverse characteristics including disease resistance, maturity classification and adaptation to the agro-ecological zones.

A split-block design with three replicates was used with sowing date as main plot and genotypes randomised within plots. The sowing depth was 3–5 cm and the seeding rate was adjusted to achieve a sowing density of 40 seeds/m^2^. Each plot was 12 m long (cut back to 10 m after emergence) and consisted of five rows at TARC and six rows at WWAI, LFS and YAI. At sowing, a *Rhizobium* group N peat-based inoculant (New Edge Microbials, Albury, Australia), was made into a water slurry and injected into the furrow at a rate of 80 L/ha. Local best management practices were used including hand chipping and applying registered herbicides, fungicides and insecticides to minimise the effects of weeds, diseases and insect pests.

### 4.3. Phenological Measurements

Phenological measurements were scored as described previously for chickpea [9,34]. Emergence date (D50%emer) was recorded as the day when 50% of the targeted population had emerged, counting plants in the inside rows in two separate m^2^ quadrats. After emergence a reference area was marked within the plots by counting 20 plants in an inside row. All subsequent phenological measurements were taken from this reference area.

In indeterminant species, determination of flowering duration can be confounded due to difficulties in establishing and capturing true flower initiation especially if severe abiotic stresses cause rapid bud and flower abortion. To account for this, this study used days to 50% flowering as a more consistent measure of flower development as it can be reliably and accurately scored. Days to 50% flowering (D50%F) were recorded as the date when 50% of the 20 plants (i.e., 10 plants) within the reference area had at least one open flower. End of flowering was recorded when flowers from all plants within the reference area had withered or dropped. Similar measurements were taken for days to pod initiation (D10%P), days to 50% podding (D50%P), and days to physiological maturity (DTPM, defined as the date when 95% of the pods in a plot changed to a yellow brown colour. These measurements were then used to calculate the vegetative (VD), flowering (FD) and podding (PD) durations. Growing degree days (GDD) were calculated from daily mean temperatures using a base temperature of 0 °C [34].

### 4.4. Statistical Analysis

Statistical analysis was done using the Restricted Maximum Likelihood (REML) spatial linear model algorithm in GenStat 20th Edition [35]. Statistical analysis was done for individual experiments (single site and year), to understand the effect of genotype, sowing date and the interaction between them. The predicted means for days to flowering generated from the REML model were used to test the environmental correlations, G × E interactions, genotype adaptability/stability using the additive main effects and multiplicative interaction (AMMI) model analysis. Furthermore, the predicted means were used to characterise the environments using the genotype main effects (G) and genotype by environment interaction (G × E) GGE model. For the AMMI and GGE models, a balanced dataset of seven genotypes common in all the experiments was used because unbalanced datasets can affect the validity of the analysis [36].

## Figures and Tables

**Table 1:** Mean daily temperature (°C) at each sowing date (SD) at Trangie Agricultural Research Centre (TARC), Wagga Wagga Agricultural Institute (WWAI), Leeton Field Station (LFS), and Yanco Agricultural Institute (YAI) in 2018 and 2019.

	2018				2019			
	SD1 (Mid April)	SD2 (Late April)	SD3 (Mid May)	SD4 (Late May)	SD1 (Mid April)	SD2 (Late April)	SD3 (Mid May)	SD4 (Late May)
TARC	19.4	16.1	14.1	17.2	21.7	18.4	17.4	8.8
WWAI	15.5	13.3	11.3	13.5	21.8	15.5	9.8	7.5
LFS/YAI	18.5	15.2	12.9	16.4	22.9	16.0	10.4	7.4

**Table 2:** Influence of sowing date on key chickpea development phases. SD = Sowing Date, Est = establishment; D50%emer = days to 50% emergence; D50%F = days to 50% flowering; D10%P = days to 10% podding; D50%P = days to 50% podding; DTPM = days to physiological maturity; VD = vegetative duration; FD = flowering duration; PD = podding duration.

Experiment	SD	Est (m^2^)	D50%emer	D50%F	D10%P	D50%P	DTPM	VD (Days)	FD (Days)	PD (Days)
TARC2018	1 (mid April)	13.3	–	99.3	108.4	121.9	180.2	–	56.0	58.3
	2 (late April)	14.0	–	97.7	108.6	117.9	168.3	–	45.0	50.4
	3 (mid May)	11.9	–	98.5	109.8	117.3	160.1	–	37.2	42.8
	4 (late May)	12.4	–	96.0	105.2	109.7	148.8	–	27.7	39.1
	*p* value	0.108		<0.001	0.276	<0.001	<0.001		<0.001	<0.001
	l.s.d. (*p* < 0.05)	ns		1.64	ns	3.02	3.88		6.81	5.99
										
TARC2019	1 (mid April)	44.2	6.1	93.1	103.5	121.1	192.1	70.3	60.5	88.6
	2 (late April)	38.4	13.4	97.1	111.1	126.8	177.1	70.4	38.5	66.0
	3 (mid May)	51.9	12.4	103.7	109.6	118.3	163.3	80.4	22.0	53.7
	4 (late May)	45.4	25.2	96.2	102.7	109.0	149.2	64.6	19.5	46.5
	*p* value	<0.001	<0.001	<0.001	<0.001	<0.001	<0.001	<0.001	<0.001	<0.001
	l.s.d. (*p* < 0.05)	3.28	0.49	3.57	3.88	2.63	1.25	6.29	7.48	5.00
										
WWAI2018	1 (mid April)	31.8	6.1	147.8	166.4	169.6	191.7	141.7	35.3	22.1
	2 (late April)	35.0	16.2	135.2	153.3	156.2	178.0	119	34.3	21.8
	3 (mid May)	37.2	22.0	125.5	140.2	142.7	164.4	103.5	31.3	21.7
	4 (late May)	34.6	26.0	118.6	128.6	131.2	151.3	92.6	25	20.1
	*p* value	0.06	<0.001	<0.001	<0.001	<0.001	<0.001	<0.001	<0.001	0.002
	l.s.d. (*p* < 0.05)	ns	0.73	0.91	0.55	0.46	0.63	1.19	0.77	0.74
										
WWAI2019	1 (mid April)	41.5	9.2	153.0	168.1	170.2	198.5	140.8	23.6	30.4
	2 (late April)	47.4	14.9	141.1	153.5	156.2	192.1	124.2	21.5	38.6
	3 (mid May)	44.3	18.2	128.6	141.0	142.8	179.4	108.7	25.8	38.4
	4 (late May)	39.0	22.4	117.3	127.6	129.0	163.8	92.7	28.0	36.2
	*p* value	<0.001	<0.001	<0.001	<0.001	<0.001	<0.001	<0.001	<0.001	<0.001
	l.s.d. (*p* < 0.05)	2.12	0.52	0.42	0.55	0.73	1.9	0.68	1.19	1.96
										
LFS2018	1 (mid April)	38.1	10.0	117.3	145.4	158.8	203.8	107.3	60.3	45.0
	2 (late April)	39.0	17.3	118.4	141.3	147.0	190.2	101.1	44.6	43.3
	3 (mid May)	35.7	20.2	118.2	132.6	137.9	175.6	98.1	30.9	37.7
	4 (late May)	36.6	24.7	110.3	123.2	127.9	162.3	85.6	26.6	34.5
	*p* value	0.229	<0.001	<0.001	<0.001	<0.001	<0.001	<0.001	<0.001	<0.001
	l.s.d. (*p* < 0.05)	ns	0.514	1.209	1.819	1.308	1.221	1.369	1.799	1.755
										
LFS2019	1 (mid April)	40.8	11.1	131.1	154.3	160.3	193.9	97.00	57.5	39.6
	2 (late April)	44.9	15.2	130.7	140.8	145.4	180.4	110.3	27.2	39.6
	3 (mid May)	40.8	18.5	121.8	128.3	132.0	167.3	97.2	25.2	39.0
	4 (late May)	46.5	29.0	112.5	117.7	122.2	154.3	78.1	21.8	36.7
	*p* value	<0.001	<0.001	<0.001	<0.001	<0.001	<0.001	<0.001	<0.001	<0.001
	l.s.d. (*p* < 0.05)	2.65	0.33	1.52	0.57	0.71	0.8	1.62	2.05	1.08
										
YAI2018	1 (mid April)	38.0	11.7	106.0	145.0	160.9	196.5	94.4	69.1	35.5
	2 (late April)	36.5	17.4	106.9	140.0	146.6	182.5	89.5	54.0	35.9
	3 (mid May)	37.7	20.3	107.6	130.0	135.3	169.0	87.3	41.0	33.7
	4 (late May)	34.8	26.0	112.4	121.7	126.3	157.1	86.4	24.1	30.8
	*p* value	0.638	<0.001	0.008	0.004	<0.001	<0.001	0.332	<0.001	0.316
	l.s.d. (*p* < 0.05)	ns	0.412	3.221	9.362	3.9	1.272	ns	2.308	ns

ns = not significant (l.s.d. not calculated).

**Table 3:** Experiment site details and overall trial management for the 2018 and 2019 chickpea experiments at Trangie Agricultural Research Centre (TARC) (31.99° S, 147.95° E), Wagga Wagga Agricultural Institute (WWAI) (35.05° S, 147.35° E), Leeton Field Station (LFS) (34.59° S, 146.36° E), and Yanco Agricultural Institute (YAI) (34.61° S, 146.41° E).

	TARC		WWAI		LFS		YAI
	2018	2019	2018	2019	2018	2019	2018
Soil type	Red chromosol	Red chromosol	Kandosol	Kandosol	Brown chromosol	Brown chromosol	Brown chromosol
Soil acidity (pHc_a_)	5.6 (10 cm)	5.9 (10 cm)	5.9 (10 cm)	5.0 (10 cm)	6.4 (10 cm)	6.0 (10 cm)	6.0 (10 cm)
Previous crop	Barley	Barley	Barley	Wheat	Barley	Barley	Bare fallow
Fertiliser	85 kg/ha of Granulock^®^ Z Extra	85 kg/ha of Granulock^®^ Z Extra	100 kg/ha of Granulock^®^ Z Soygran	100 kg/ha of Granulock^®^ Z Soygran	Zero	55 kg/ha Utiliser pulse mix	80 kg/ha of Energiser Plus
	(N 9.86: P 16.83: K 0.0: S 4.59: Zn 1.70)	(N 9.86: P 16.83: K 0.0: S 4.59: Zn 1.70)	(N 5.5: P 15.3: K 0.0: S 7.5)	(N 5.5: P 15.3: K 0.0: S 7.5)		(N 13.5: P 13.5: K 0.0: S 9.5)	
Pre-sowing watering	40 mm (1 event)	175 mm (6 events)	Zero	Zero	220 mm (flood)	200 mm (flood)	77 mm (overhead)
In-crop watering	85 mm (5 events)	87 mm (4 events)	10 mm (overhead)	15 mm (SD1)	24 mm (overhead)	NA	64 mm (overhead)
Growing season rainfall (Apr–Oct)	137 mm	45 mm	153 mm	193 mm	87 mm	160 mm	87 mm

**Table 4:** Chickpea genotypes and their classification/characteristics evaluated in the 2018 and 2019 seasons.

Genotype	Type	Maturity	2018	2019
CICA1521	Desi	Early	Yes	Yes
PBA Striker	Desi	Early	Yes	Yes
PBA Slasher	Desi	Mid	Yes	Yes
PBA Boundary	Desi	Mid–Late	Yes	Yes
Neelam	Desi	Mid	Yes	No
PBA HatTrick	Desi	Mid	No	Yes
Genesis 079	Kabuli	Early	Yes	Yes
Genesis 090	Kabuli	Mid–Late	Yes	Yes
Genesis Kalkee	Kabuli	Late	Yes	Yes

## Supplementary Materials

The following are available online at https://www.mdpi.com/2223-7747/9/10/1257/s1, Figure S1: title, Table S1: title, Video S1: title. Supplementary Materials: The following are available online at https://www.mdpi.com/2223-7747/9/10/1257/s1, as excel spreadsheets. Table S1: Complete REML statistical analysis results showing effect of sowing time, genotype and the interaction between the two (G × E) for TARC in 2018 and 2019. Table S2: Complete REML statistical analysis results showing effect of sowing time, genotype and the interaction between the two (G × E) for WWAI in 2018 and 2019. Table S3: Complete REML statistical analysis results showing effect of sowing time, genotype and the interaction between the two (G × E) for LFS in 2018 and 2019. Table S4: Complete REML statistical analysis results showing effect of sowing time, genotype and the interaction between the two (G × E) for YAI in 2018 and 2019.
